# Validation of the pooled cohort risk score in an Asian population – a retrospective cohort study

**DOI:** 10.1186/1471-2261-14-163

**Published:** 2014-11-20

**Authors:** Yook Chin Chia, Hooi Min Lim, Siew Mooi Ching

**Affiliations:** Department of Primary Care Medicine, University of Malaya Primary Care Research Group (UMPCRG), Faculty of Medicine, University of Malaya, 50603 Kuala Lumpur, Malaysia; Curtin Health Innovation Research Institute, Faculty of Health Sciences, Curtin University, GPO Box U1987, 6845 Perth, WA Australia; Department of Family Medicine, Faculty of Medicine and Health Sciences, Universiti Putra Malaysia, 43400 Serdang, Malaysia; Department of Gerontology, Universiti Putra Malaysia, 43400 Serdang, Malaysia

**Keywords:** Pooled cohort risk score, Atherosclerotic cardiovascular disease, Validation, Asian population, Framingham risk score, Cardiovascular events, Primary care, Retrospective cohort, Malaysia

## Abstract

**Background:**

The Pooled Cohort Risk Equation was introduced by the American College of Cardiology (ACC) and American Heart Association (AHA) 2013 in their Blood Cholesterol Guideline to estimate the 10-year atherosclerotic cardiovascular disease (ASCVD) risk. However, absence of Asian ethnicity in the contemporary cohorts and limited studies to examine the use of the risk score limit the applicability of the equation in an Asian population. This study examines the validity of the pooled cohort risk score in a primary care setting and compares the cardiovascular risk using both the pooled cohort risk score and the Framingham General Cardiovascular Disease (CVD) risk score.

**Methods:**

This is a 10-year retrospective cohort study of randomly selected patients aged 40–79 years. Baseline demographic data, co-morbidities and cardiovascular (CV) risk parameters were captured from patient records in 1998. Pooled cohort risk score and Framingham General CVD risk score for each patient were computed. All ASCVD events (nonfatal myocardial infarction, coronary heart disease (CHD) death, fatal and nonfatal stroke) occurring from 1998–2007 were recorded.

**Results:**

A total of 922 patients were studied. In 1998, mean age was 57.5 ± 8.8 years with 66.7% female. There were 47% diabetic patients and 59.9% patients receiving anti-hypertensive treatment. More than 98% of patients with pooled cohort risk score ≥7.5% had FRS >10%. A total of 45 CVD events occurred, 22 (7.2%) in males and 23 (3.7%) in females. The median pooled cohort risk score for the population was 10.1 (IQR 4.7-20.6) while the actual ASCVD events that occurred was 4.9% (45/922). Our study showed moderate discrimination with AUC of 0.63. There was good calibration with Hosmer-Lemeshow test *χ*2 = 12.6, *P* = 0.12.

**Conclusions:**

The pooled cohort risk score appears to overestimate CV risk but this apparent over-prediction could be a result of treatment. In the absence of a validated score in an untreated population, the pooled cohort risk score appears to be appropriate for use in a primary care setting.

## Background

The American College of Cardiology (ACC) and American Heart Association (AHA) released a new guideline on blood cholesterol management in November 2013. This guideline introduced the new pooled cohort risk equation to estimate 10-year atherosclerotic cardiovascular disease (ASCVD) risk, which includes nonfatal myocardial infarction (MI), coronary heart disease (CHD) death, nonfatal and fatal stroke [1]. The pooled cohort risk equation was derived from four major population-based cohort studies in the United States involving white and black Americans and this new risk tool was further validated by two external cohorts: Reasons for Geographic and Racial Differences in Stroke (REGARDS) and Multi-Ethnic Study of Atherosclerosis (MESA) [2, 3]. This pooled cohort risk score serves as a guide to help clinician in deciding statin initiation for patients with mildly elevated CV risk. Statin is recommended as primary prevention for those non-diabetic patients with LDL 90-189 mg/dl and pooled cohort risk score ≥7.5%.

Opinions and debates have arisen since the introduction of the pooled cohort risk score. The applicability of the pooled cohort risk score is being questioned because it is perceived to overestimate CV risk due to the lower cut-off point of 7.5% and that more patients will need to be treated with statins if the pooled cohort risk score is applied [4–6].

Absence of Asian ethnicity in the contemporary cohorts and external cohorts limit the applicability of the pooled cohort risk score in the Asian population. Until recently very few studies examined the use of pooled cohort risk score in different populations. Hence, our aim was to validate the use of the pooled cohort risk equation in an Asian population. We also compared the CV risk of our population using both the pooled cohort risk score and the Framingham General CVD risk score.

## Methods

### Setting

This study is a 10-year retrospective cohort study of randomly selected patients registered with an outpatient primary care clinic of University Malaya Medical Centre (UMMC). The hospital is located in Kuala Lumpur, the capital of Malaysia. The main ethnic groups in this population are Malay, Chinese and Indian. The outpatient clinic is under the Department of Primary Care Medicine which is run by 14 family medicine specialists and 30 vocational trainees in family medicine. Ethics approval was obtained from the Ethics Committee of the institution.

### Study population

**Figure 1:**
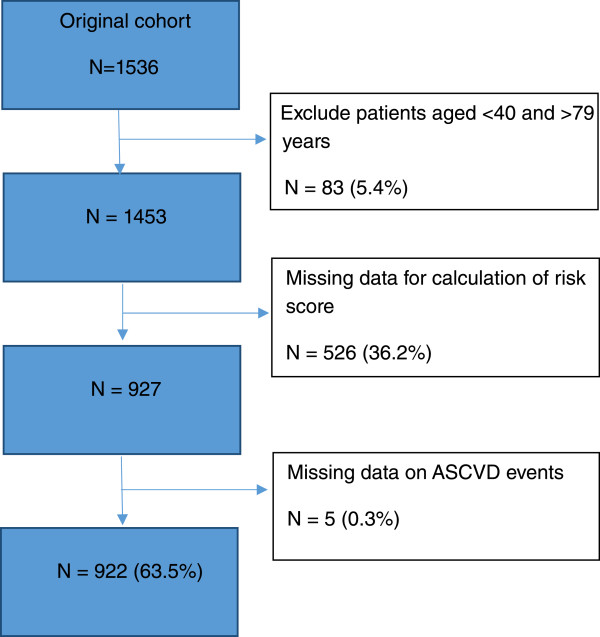
**Flowchart of patients included in the analysis.**

There were 1536 patients in our original cohort. We excluded patients aged <40 or aged >79 (n = 83) as this was out of the age range of the pooled cohort risk score calculator. Out of 1453 patients, 526 (36.2%) patients were also excluded as they did not have all the variables needed to calculate the pooled cohort risk score. Another 5 patients was excluded as we could not ascertain their CVD status by the end of 2007. Hence a total of 922 patients (63.5%) were eligible for analysis (Figure 1). Our follow-up rate over the 10-years was 95% (871/922) with only 51 (5%) patients who did not come for follow up.

### Inclusion criteria

Adults aged 40–79 without clinical ASCVD who were already registered in our centre in 1998.

### Exclusion criteria

Patients who did not have all the variables to calculate the pooled cohort risk score at baseline were excluded. We also excluded patients with missing data on the ASCVD event.

### Data collection

Random numbers were generated by computer based on the patients’ registration number with the clinic. Baseline data was collected in 1998 and follow-up data collected in 2007, a 10-year interval. We extracted the patients’ information from their paper-based records manually. Socio-demograhic data and co-morbidities were recorded.

A pooled cohort risk score for each patient was computed using the online Pooled Cohort Risk Equation provided by the American Heart Association [7]. For the variable race, we used ‘White or others’ for our study population. The Framingham risk score (FRS) for 10-year risk of cardiovascular disease (CVD) was also calculated based on the Framingham General CVD risk score chart [8]. All the variables needed for calculation of both the scores are shown in Table 1. ASCVD events in the 10-year period (1998–2007) were captured. ASCVD event is defined as nonfatal myocardial infarction (MI), CHD death, nonfatal and fatal stroke [1]. ASCVD events in our cohort were based on the clinicians’ diagnosis supported by the relevant investigations. For those who defaulted and did not complete their subsequent 10 year follow-up at our clinic (n = 31) we traced and examined their case records with the main hospital to determine their CVD outcome. For those patients who did not attend our clinics or hospital after being entered into our study, we called the patient or their family individually (n = 20) to ascertain their CVD status. We were not able to ascertain CVD outcome in only 5 patients.

**Table 1:** **Variables for pooled cohort risk score and Framingham general CVD risk score**

	Pooled cohort risk score	Framingham general CVD risk score
**Variables**	Sex	Sex
	Age	Age
	Race	-
	Total cholesterol (mg/dl)	Total cholesterol (mg/dl)
	HDL cholesterol (mg/dl)	HDL cholesterol (mg/dl)
	Systolic blood pressure (mmHg)	Systolic blood pressure (mmHg)
	Treatment for high blood pressure	Treatment for high blood pressure
	Diabetes	Diabetes
	Smoker	Smoker
**CVD events**	Nonfatal myocardial infarction	Nonfatal myocardial infarction
	CHD death	CHD death
	Nonfatal stroke	Angina
	Fatal stroke	Cardiac failure
		Nonfatal stroke
		Fatal stroke
		Peripheral arterial disease

CVD Cardiovascular Disease; HDL high density lipoprotein; CHD cardiovascular heart disease.

Blood Pressure (BP) was measured by our attending doctor using a mercury sphygmomanometer as part of the daily routine practice. Diagnosis of hypertension is made in accordance with standard recommendations i.e. BP ≥140/90 mmHg based on at least 2 blood pressure measurements at least 2 weeks apart [9]. The use of anti-hypertensive agents was recorded as well. Diabetes mellitus was defined as documented by the attending physician or the use of hypoglycaemic agents or both. Smokers were defined if they were still smoking currently. Non-smokers were those who never smoked or currently not smoking regardless of when they had stopped smoking. Total cholesterol, LDL and HDL cholesterol were also collected. All blood tests were performed in our teaching hospital’s laboratory which is certified by the Royal College of Pathologists of Australasia standards. Statin use in 1998 and 2007 was recorded.

### Statistical analysis

All statistical analysis was done using the Statistical Package for Social Sciences (SPSS version 15). Categorical data are reported as proportions (percentage). Mean was used for continuous variables that were normally distributed. Median and interquartile range were used for variables that were not normally distributed.

### Discrimination

Discrimination is defined as the ability of a risk prediction model to accurately rank order individuals (i.e. are individuals with higher predicted risk more likely to have events.) We used receiver operating characteristic (ROC) curve to determine the discriminative power of the pooled cohort risk score. The area under the curve (AUC), also known as c-index, was used as a measure of how well the pooled cohort risk score can discriminate. Discrimination is defined as good when the c index is closer to 1 whereas a value of 0.5 implies that the risk score tool is no better than chance [10].

### Calibration

Calibration was used to assess whether the observed 10-year ASCVD events differed significantly from predicted [10]. The calibration of the pooled cohort risk score was determined using Hosmer-Lemeshow test [11]. A *χ*2 value of greater than 20 or a *p* value of less than 0.05 indicates poor calibration.

### Net reclassification Index (NRI)

NRI was used to quantify improvement offered by pooled cohort risk score compared to Framingham General CVD risk score. We adopted the approach proposed by Pencina et al. for the NRI analysis [12].

## Results

A total of 922 patients were eligible for this study. Table 2 shows the clinical characteristics of the study population at baseline in 1998. Overall, the mean age of the population was 57.5 ± 8.8 years and about two-thirds of the population was female (66.7%, n = 615). The major ethnic group was Chinese (46.1%, n = 425), followed by Indian (29.8%, n = 275) and Malay (22.7%, n = 210). Nearly half of the patients were diabetic (47%). 87.5% (n = 371) of these diabetic patients were receiving treatment with either oral hypoglycaemic agents and/or insulin. The mean systolic BP was 140.9 ± 18.6 mmHg and more than half of the population was treated with anti-hypertensive agents. Only 9.7% of the population (n = 90) received statin therapy in 1998. We also compared the baseline characteristics for those who were entered into our analysis to the patients who were excluded. Basically there was no substantial difference between their baseline characteristics.

**Table 2:** **Comparison of CV risk factors in 1998 and 2007**

Clinical characteristics	1998	2007
**Age, year (mean)**	57.5 ± 8.8	67.5 ± 8.8
**Sex, female (n, %)**	615 (66.7)	615 (66.7)
**Ethnicity (n, %)**		
**- Malay**	210 (22.7)	210 (22.7)
**- Chinese**	425 (46.1)	425 (46.1)
**- Indian**	275 (29.8)	275 (29.8)
**- Others**	12 (1.4)	12 (1.4)
**Diabetes mellitus (n, %)**	424 (47.0)	528 (57.3)
**- HbA1c, % (mean)**	7.7 ± 1.8	7.5 ± 1.6
**- DM controlled with HbA1c ≤6.5% (n, %)**	110 (11.9)	163 (17.7)
**Systolic BP, mmHg (mean)**	140.9 ± 18.6	135.1 ± 16.6
**- Use of anti-hypertensive agents (n, %)**	552 (59.9)	760 (82.4)
**- BP controlled SBP ≤140 mmHg (n, %)**	568 (61.6)	681 (73.9)
**- RAS blocker use (n, %)**	66 (7.2)	371 (40.2)
**Total cholesterol, mg/dl (mean)**	234.8 ± 42.3	190.9 ± 37.2
**HDL cholesterol, mg/dl (mean)**	47.6 ± 14.2	49.6 ± 12.5
**LDL cholesterol, mg/dl (mean)**	143.1 ± 40.7	115.5 ± 31.6
**Statin use in 1998 (n, %)**	90 (9.7)	587 (63.7)
**Smoker (n, %)**	56 (6.1)	56 (6.1)

Mean ± Standard deviation.

CV cardiovascular, DM diabetes mellitus, BP blood pressure, SBP systolic blood pressure, RAS renin-angiotensin system, HDL high density cholesterol, LDL low density cholesterol.

Table 2 also shows the change in CVD risk factors at the end of 10 years. The number of diabetic patients increased from 47.0% to 57.3% but HbA1c control improved from a mean of 7.7% to 7.5%. The mean systolic blood pressure was reduced by 5.8 mmHg, from 140.9 mmHg to 135.1 mmHg. The number of patients who had controlled BP with systolic BP ≤140 mmHg increased from 61.6% to 73.9%. There was an increase in the use of renin-angiotensin system (RAS) blockers from 7.2% to 40.2%. The number of patients receiving statin increased significantly from 9.7% to 63.7% in 2007. There was also improvement in the lipid profile of patients at the end of 10 years compared to baseline. Mean of total cholesterol was reduced from 234.8 mg/dl to 190.9 mg/dl while the mean HDL increased from 47.6 mg/dl to 49.6 mg/dl.

Table 3 compares the 10-year CV risk of our population at baseline using both the FRS and the pooled cohort risk score. For those with pooled cohort risk score of 7.5-9.9%, 98.8% of them have FRS ≥10%. Whereas in those patients with pooled cohort risk score 10–19.9% and ≥20%, 99.6% of them have FRS ≥10%. Hence, we conclude that more than 98% of patients with pooled cohort risk score ≥7.5% have a FRS of >10%. In those with pooled cohort risk score of <7.5%, two-thirds have FRS of >10%. Only 13.4% (124/922) have low risk in both risk scores.

**Table 3:** **Comparison of 10-year cardiovascular risk in 1998 between the pooled cohort risk score and Framingham general CVD risk score**

10-year CVD risk (Framingham general CVD risk score)	10-year ASCVD risk (pooled cohort risk score)				
	(N = 922)				
	<7.5%	7.5-9.9%	10.0-19.9%	≥20%	Total
	N (% of total N)	N (% of total N)	N (% of total N)	N (% of total N)	
**<10%**	124 (34.0)	1 (1.2)	1 (0.4)	1 (0.4)	128
**10-20%**	197 (53.7)	35 (40.7)	44 (19.3)	2 (0.8)	278
**>20%**	45 (12.5)	50 (58.1)	183 (80.3)	238 (98.8)	520
**Total**	366	86	228	241	922

ASCVD: atherosclerosis cardiovascular disease; CVD cardiovascular disease.

Table 4 shows the comparison of predicted and observed pooled cohort risk score in 1998 and ASCVD events in the 10-year interval from 1998 to 2007 in our primary care patients. The median pooled cohort risk score for the study population was 10.1% (IQR 4.7-20.6). The actual number of ASCVD events that occurred in the 10 years was 45 (45/922 = 4.9%) while the predicted was 93. In men, their median pooled cohort risk score was 21.1% while the events that occurred was 22 (7.2%). In women, their median pooled cohort risk score 6.7% while the event that occur was 23 (3.7%). Similarly the events rate was twice as high in men (7.2%) than women (3.7%). Interestingly, we noticed that 8 (2.2%) ASCVD events occurred in those patients with pooled cohort risk score ≤7.5% (N = 367). We analysed the CV risk for this group of patients and found out that 36% had DM and 51% hypertension. Over the 10-year period, the prevalence of DM and hypertension increased to 49% and 80.9% respectively. The statin use for this low risk group of patients was increased from 10.4% to 68.1%.

**Table 4:** **Comparison of predicted and observed pooled cohort risk score in 1998 and ASCVD events in a 10-year interval (1998–2007)**

Pooled cohort risk score in 1998,%	Total, N	Observed ASCVD event (n, %)	Predicted ASCVD Events (n, %)
**All adults**			
**Median score: 10.1%**	**922**	**45 (4.9)**	**93 (10.1)**
**(95% CI 4.7-20.6)**			
**-** <7.5%	367	8 (2.2)	14 (3.8)
**-** 7.5-9.9%	86	6 (7.0)	7 (8.4)
**-** 10-19.9%	228	12(5.3)	32 (13.9)
**-** ≥20%	241	10(7.9)	73 (30.5)
**Male**			
**Median score: 21.1%**	**307**	**22 (7.2)**	**64 (21.1)**
**(95% CI 12.0-31.9)**			
**-** <7.5%	32	0	1 (4.5)
**-** 7.5-9.9%	19	1 (5.3)	2 (8.2)
**-** 10-19.9%	92	6 (6.5)	13 (14.3)
**-** ≥20%	164	15 (9.1)	51 (31.1)
**Female**			
**Median score: 6.7%**	**615**	**23 (3.7)**	**41 (6.7)**
**(95% CI 3.3-13.0)**			
**-** <7.5%	335	8 (2.4)	12 (3.6)
**-** 7.5-9.9%	67	5 (7.5)	6 (8.4)
**-** 10-19.9%	136	6 (4.4)	18 (13.5)
**-** ≥20%	77	4 (5.2)	22 (28.2)

CI = Confidence Interval.

**Figure 2:**
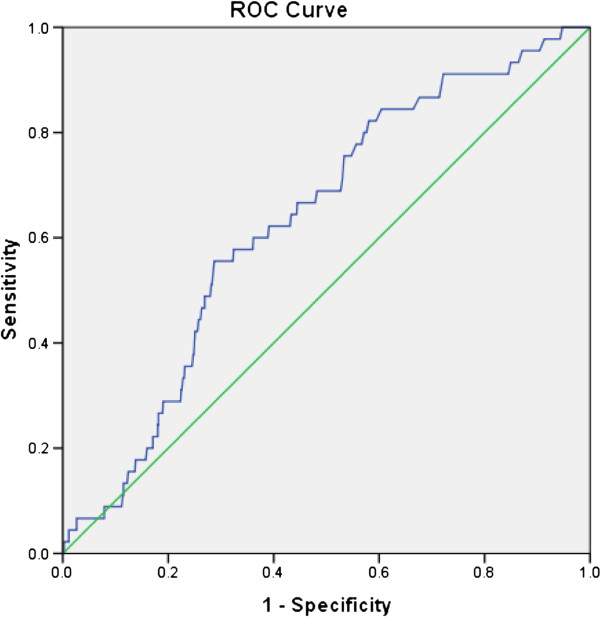
**ROC and AUC for pooled cohort risk score.**

The AUC for pooled cohort risk score was 0.63 showing moderate discrimination as shown in Figure 2. The calibration for pooled cohort risk score was good as the Hosmer-Lemeshow test result was *χ*2 = 12.6, *P* = 0.12. We also calculated the Net Reclassification Index (NRI) and the NRI is 3.1% (p = 0.001). This indicates that the pooled cohort risk score provided 3.1% more net reclassification improvement.

Table 5 shows the comparison between pooled cohort risk score and ASCVD events according to ethnicity. The pooled cohort risk score discriminates moderately well for Malay race (AUC 0.73, p = 0.011) but not for the Chinese and Indian.

**Table 5:** **Comparison pooled cohort risk score and ASCVD events according to ethnicity**

	Pooled cohort risk score (%)	ASCVD event		AUC (95% CI)	p-value
		Observed	Predicted		
		N (%)	N		
Overall (N = 922)	10.1	45 (4.9)	93	0.632 (0.557,0.70)	0.003
Malay (N = 210)	7.9	10 (4.8)	17	0.737 (0.641,0.834)	0.011
Chinese (N = 425)	10.8	21 (4.9)	46	0.625 (0.512,0.737)	0.054
Indian (N = 275)	10.0	14 (5.1)	28	0.576 (0.417,0.736)	0.335

AUC = Area under curve.

## Discussion

A very recent study validated the pooled cohort risk score in a US population and found it to work very well [2]. Our study showed that the pooled cohort risk score has moderate discrimination and good calibration in an Asian population. One possible explanation of our finding of only moderate discrimination could be because of the high prevalence of patients with CV risk factors (i.e. the high prevalence of diabetes and hypertension seen) and therefore a clustering of patients with higher risk and fewer patients with lower risk. Hence in a general population of subjects with a wider range of CV risk, this risk prediction model may give a better discrimination.

However, the number of observed events was fewer than predicted. This apparent over-estimation could be because of treatment which would result in a reduction of ASCVD events. As seen in our study, the number of patients receiving statin therapy increased from 9.7% (n = 90) to 63.7% (n = 587) over the 10-year period. Besides the increase in statin use other CV risk factors were also improved where the mean systolic blood pressure, mean HbA1c, total and LDL-cholesterol were all reduced over the 10-year period. Control rate for blood pressure and diabetes as well as use of RAS blockers also increased [13]. Studies have shown that a reduction of 10 mmHg of blood pressure can result in a reduction of 25% of CHD, 45% of stroke and 55% of heart failure [14, 15]. Furthermore, a 1% decrease in HbA1c level can lead to a reduction of fatal and non-fatal MI by 14%, peripheral vascular disease by 43% and heart failure by 16% [16]. For LDL cholesterol level, every 38 mg/dl of LDL reduction associated with 20% reduction of CVD events [17]. In view of the treatment given and the control rates achieved, there would be substantial improvement of all the CV risk factors in our study population over the 10-year period, leading to significant reduction of ASCVD events.

One of the reasons the pooled cohort risk score was introduced is because it is derived from a more diverse population when compared to FRS which is based on a more homogenous cohort. While the pooled cohort risk score has not been widely validated yet, the Framingham General CVD risk score has been well studied even outside the US [18–23]. However, NRI of 3.1% in our study showed there was no substantial improvement of the pooled cohort risk score over the FRS suggesting that both risk scores are equivalent.

There is general concern that using the lower threshold of 7.5% based on the pooled cohort risk score to determine the need for statin therapy will mean that more patients will be treated with statin [10]. However some reports suggest otherwise [24, 25]. Furthermore, our study showed that in those with pooled cohort risk score of 7.5-9.9%, almost all had a FRS of >10% suggesting that this new AHA/ACC recommendation may be appropriate.

The proportion of patients with low risk in our study is small because our cohort is made up of actual patients whose CV risks are expected to be higher and hence there will be fewer people with low risk in our cohort. However, the individuals that were identified as low risk by pooled cohort risk score did actually have ASCVD events. This group of low risk patients was relatively younger with mean age of 50.8 years. Even though their risks were calculated to be low at baseline, some of them did have diabetes (32.7%) and 51% have hypertension. Furthermore these patients’ risk increased over the 10-year period, with aging and with more of them developing diabetes (49%) and hypertension (80.6%). Statin use also increased amongst these low risk patients suggesting that they developed dyslipidaemia along the way thus increasing their risk for CV events.

Interestingly, in those patients with pooled cohort risk score <7.5%, about two-thirds of these patients have a FRS of >10%. This discrepancy could be because the FRS predicts not just only fatal and non fatal stroke, nonfatal MI and CHD death used in the pooled cohort risk score, but also include angina, cardiac failure and peripheral arterial disease. Hence the FRS score would appear magnified when compared to pooled cohort risk score.

### Strengths and limitations

Our long study period allowed us to obtain data on ASCVD events over 10 years. This is in accordance with the pooled cohort risk equation which was designed to estimate a 10-year ASCVD risk. Our study was conducted in a primary care setting where most patients have not had any ASCVD events yet. And this is the group of patients who will be the main target for risk stratification so that primary prevention can be initiated appropriately. Besides that, we were able to compare the characteristics of those with missing data and those included in the analysis and found no clinical differences between them, suggesting there was no substantial bias.

As this is a retrospective cohort study, recall bias in the actual CV events may occur especially for those patients who defaulted follow-up in our centre and therefore without proper documentation of events.

## Conclusion

In our study, when compared to using the FRS, the pooled cohort risk score using a lower threshold of 7.5% risk does not appear to overestimate CV risk. However the pooled cohort risk score appears to overestimate absolute CV risk as the observed events are less than predicted. This apparent over-prediction could be the result of treatment. Hence, in the absence of validation of the pooled cohort risk score in an untreated population, the pooled cohort risk score is appropriate for use in a primary care setting.

## Abbreviations

ASCVD: Atherosclerosis cardiovascular disease
ACC/AHA: American College of cardiology and American Heart Association
CV: Cardiovascular
HDL: High density lipoprotein
LDL: Low density lipoprotein
DM: Diabetes mellitus
BP: Blood pressure
RAS: Renin-angiotensin system
FRS: Framingham risk score risk
MI: Myocardial infarction
CHD: Coronary heart disease.
